# The Response Regulator Slr1588 Regulates *spsA* But Is Not Crucial for Salt Acclimation of *Synechocystis* sp. PCC 6803

**DOI:** 10.3389/fmicb.2017.01176

**Published:** 2017-06-26

**Authors:** Kuo Song, Martin Hagemann, Xiaoming Tan, Xuefeng Lu

**Affiliations:** ^1^Key Laboratory of Biofuels, Shandong Provincial Key Laboratory of Synthetic Biology, Qingdao Institute of Bioenergy and Bioprocess Technology, Chinese Academy of SciencesQingdao, China; ^2^Department of Plant Physiology, Institute of Biological Sciences, University of RostockRostock, Germany; ^3^Laboratory for Marine Biology and Biotechnology, Qingdao National Laboratory for Marine Science and TechnologyQingdao, China

**Keywords:** *slr1588*, *Synechocystis*, *spsA*, glucosylglycerol, sucrose, *ggpP*, response regulator, transcriptional regulation

## Abstract

Cyanobacterial sucrose biosynthesis is stimulated under salt stress, which could be used for biotechnological sugar production. It has been shown that the response regulator Slr1588 negatively regulates the *spsA* gene encoding sucrose-phosphate synthase and mutation of the *slr1588* gene also affected the salt tolerance of *Synechocystis* (Chen et al., 2014). The latter finding is contrary to earlier observations (Hagemann et al., 1997b). Here, we observed that ectopic expression of *slr1588* did not restore the salt tolerance of the *slr1588* mutant, making the essential function of this response regulator for salt tolerance questionable. Subsequent experiments showed that deletion of the entire coding sequence of *slr1588* compromised the expression of the downstream situated *ggpP* gene, which encodes glucosylglycerol-phosphate phosphatase for synthesis of the primary osmolyte glucosylglycerol. Mutation of *slr1588* by deleting the N-terminal part of this protein (Δ*slr1588*-F976) did not affect *ggpP* expression, glucosylglycerol accumulation as well as salt tolerance, while the mutation of *ggpP* resulted in the previously reported salt-sensitive phenotype. In the Δ*slr1588*-F976 mutant *spsA* was up-regulated but sucrose content was lowered due to increased invertase activity. Our results reveal that Slr1588 is acting as a repressor for *spsA* as previously suggested but it is not crucial for the overall salt acclimation of *Synechocystis*.

## Introduction

Increasing concerns about growing human populations, resource use and proposed climate change scenarios promoted the development of sustainable methods for biomass and energy production. One approach aims to use microalgae and cyanobacteria as green cell factories for the production of valuable products. Genetic engineering of cyanobacteria was well established and allowed the generation of strains producing various biofuels (Kopka et al., 2017). Carbohydrates such as sucrose are also interesting products (Hays and Ducat, 2015), since they can be directly used for human nutrition or for feeding heterotrophic bacteria in biotechnological co-cultivation approaches (Ducat et al., 2012). Metabolic engineering approaches combining overexpression of sucrose synthesis enzymes and heterologous sucrose exporters generated cyanobacterial strains that showed great potentials in sucrose production (Ducat et al., 2012; Song et al., 2016).

Naturally, cyanobacterial sucrose accumulation is part of their acclimation to high salt concentrations. To cope with salt stress, cyanobacteria developed two main strategies: actively exporting inorganic ions (e.g., Na^+^) and accumulating organic osmolytes such as sucrose, trehalose, glucosylglycerol (GG), glycine betaine (Hagemann, 2011) or homoserine betaine (Pade et al., 2016). A high body of research on salt acclimation and the biosynthesis of osmolytes was performed using the model cyanobacterium *Synechocystis* sp. PCC 6803 (hereafter *Syn6803*). In *Syn6803*, sucrose is transiently accumulated as secondary osmolyte additionally to the primary osmolyte GG (Reed and Stewart, 1985; Marin et al., 2004). As in plants, sucrose is synthesized from UDP-glucose and fructose 6-phosphate by a two-step pathway consisting of sucrose-phosphate synthase (encoded by *spsA*) (Hagemann and Marin, 1999) and sucrose-phosphate phosphatase (encoded by *spp*) among cyanobacteria (Lunn, 2002; Fieulaine et al., 2005). Invertase encoded by *invA* is responsible for sucrose degradation (Vargas et al., 2003). In our previous work, we found that *spsA* overexpression leads to a threefold increase in sucrose accumulation of *Syn6803* under salt-stress conditions. This finding provided evidence that SpsA catalyzes the limiting step of sucrose accumulation (Du et al., 2013). The *spsA* gene of *Syn6803* was found to be induced after salt shock and sucrose was mainly accumulated at the early stage of salt acclimation (Desplats et al., 2005). However, regulatory mechanisms and signal transduction pathways for both, *spsA* transcription and sucrose biosynthesis, are not well understood among cyanobacteria.

Two-component signal transduction systems, which typically consist of a sensory histidine kinase (Hik) and a response regulator (Rre), play important roles in responding to stresses in many microorganisms (Stock et al., 1989). By screening a library of 43 Hik mutants using DNA microarray analysis, four Hiks, namely Hik16, Hik33, Hik34, and Hik41, were identified to perceive and transduce the salt signal. These Hiks regulated approximately 20% of the salt-inducible genes (Marin et al., 2003). Subsequently, some Hik-Rre pairs were identified to be involved in perception of salt stress and signal transduction (Shoumskaya et al., 2005). However, these studies did not provide evidence that Hik-Rre systems directly regulate the *spsA* transcription in *Syn6803* under salt stress. The gene *slr1588* that encodes an orphan Rre of *Syn6803* (Rre39) was reported to directly regulate accumulation of salt-stress proteins including SpsA (Chen et al., 2014). These authors showed that the *spsA* gene was highly expressed at both, RNA and protein levels, under salt-stress condition after complete deletion of *slr1588*. They also conducted electrophoretic mobility shift assays showing that the recombinant Slr1588 protein bound directly to the upstream region of *spsA* gene *in vitro*. Consistent with the regulatory role of Slr1588 on many salt-regulated proteins, the *slr1588*-null mutant showed a salt-sensitive phenotype, i.e., it could not grow in media supplemented with 4% of NaCl (Chen et al., 2014). However, this finding is inconsistent with an earlier report (Hagemann et al., 1997b), in which the *slr1588* mutant did not show changed salt resistance. Interestingly, the *slr1588* gene is situated upstream of two other genes with important functions in salt acclimation of *Syn6803*. The first downstream gene *slr0746* (*ggpP*) encodes the GG-phosphate phosphatase catalyzing the second step in GG synthesis (Hagemann et al., 1997b), whereas the second gene *slr0747* (*ggtA*) encodes the ATP-binding subunit of the ABC-transporter for GG, trehalose, and sucrose (Hagemann et al., 1997a). It has been reported that the *ggpP* mutant of *Syn6803* is sensitive to salt stress (Hagemann et al., 1997b), whereas the *ggtA* mutant grew like wild type (WT) in NaCl-supplemented BG11 medium (Hagemann et al., 1997a).

Due to the inconsistency in previous reports on the role of the Rre Slr1588 in salt acclimation of *Syn6803*, we re-evaluated its functions using a set of newly generated mutants including complementation of the *slr1588*-null mutant. The salt tolerances, osmolyte accumulation and expression levels of selected genes were analyzed in these strains under salt-stress conditions. These experiments revealed the actual role of Slr1588 in *spsA* expression regulation but not in salt tolerance of *Syn6803*.

## Materials and Methods

### Chemicals and Reagents

Chemicals were obtained from the Sinopharm Chemical Reagent Company (China). Taq and Pfu DNA polymerases were purchased from Transgen Biotech (China). Restriction endonucleases were purchased from ThermoFisher (United States). The kits used for molecular cloning were purchased from Omega (United States). GG standard was obtained from the Bitop company (Germany).

### Plasmids and Strains Construction

A modified fusion polymerase chain reaction (PCR) method was employed for the construction of gene knockout constructs (Wang et al., 2002). Briefly, for gene targeting, we used three sets of primers (Supplementary Table S3) to amplify a kanamycin resistance cassette from pET28a (Novagen) and two arms of the targeted genes from the genome of *Syn6803*. The three DNA fragments were denatured, annealed, and extended. The resulting mixture was subsequently amplified with a set of nested primers (Supplementary Table S3). Then, the fused PCR fragment was ligated into *pEasy*-Blunt vector (TransGen Biotech, China). The resulting plasmids (Supplementary Table S2) were used for transforming the WT strain (glucose-tolerant) of *Syn6803* for gene targeting.

To generate plasmids used for complementation of the *slr1588* mutant, restriction sites of *Nde*I and *Xho*I were added to the ends of *slr1588* ORF by PCR (Supplementary Table S3). The PCR products were cut by *Nde*I and *Xho*I and ligated into the pXT37b (Tan et al., 2011), which was cut with the same enzymes, resulting in plasmid pSK005. The P*_cpcB_* promoter fragment was cut from pXX47 (Zhu et al., 2015) using *Kpn*I/*Nde*I, and ligated into the same sites of pSK005, resulting in pSK008. The gene *slr1588* including 230 bp upstream region, which is supposed to contain its native promoter, was amplified using primers pSK013-1 and pSK013-2 (Supplementary Table S3) and ligated into the *Kpn*I/*Xho*I sites of pXT37b, resulting in pSK013. These three plasmids were used for transforming the *slr1588* mutant.

### Transformation of *Synechocystis*

Natural transformation of *Syn6803* was performed according to published procedures (Williams, 1988). Briefly, 1 mL of exponentially growing cells was harvested. The cells were washed twice with fresh BG-11 (Rippka et al., 1979) medium. A 400 μL aliquot of cultures at a density of 1 × 10^9^ cells mL^-1^ was mixed with plasmid DNA at a final DNA concentration of 10 μg mL^-1^. After incubation under illumination of approximately 50 μmol photons m^-2^ s^-1^ at 30°C for 5 h, the cell suspension was spread onto membrane filters resting on BG11 agar plates (without antibiotic) for 1 day. The filters were then placed on fresh BG11 agar plates containing antibiotics at the appropriate concentrations. After 1–2 weeks of incubation at 30°C under illumination of approximately 50 μmol photons m^-2^ s^-1^, single colonies were streaked onto antibiotic-containing agar plates. Subsequently, selected clones were grown in liquid BG11 medium supplemented with antibiotics. The complete segregation of the mutant genotypes was confirmed by PCR and sequence analysis. The confirmed *Syn6803* mutants were used for further analysis. Mutant strains and related genotypes are listed in Supplementary Table S1, plasmids and its functions are listed in Supplementary Table S2, while PCR primers used for mutant construction and validation are listed in Supplementary Table S3.

### Cultivation and Salt Shock of *Synechocystis* Strains

The cyanobacteria were routinely cultivated on BG11 plates with appropriate antibiotics (20 and 30 μg mL^-1^ for spectinomycin and kanamycin, respectively), at 30°C, under illumination of approximately 50 μmol photons m^-2^ s^-1^. For evaluation of salt tolerances of *Syn6803* strains, they were inoculated at an initial OD_730_ of ∼0.05 in 50 mL flasks, containing 20 mL fresh BG11 medium supplemented with 4% (684 mM) NaCl and appropriate antibiotics. The cells were cultivated at 30°C with shaking (150 rpm) and constant white light illumination (50 μmol photons m^-2^ s^-1^). The growth was monitored by measuring the optical density (OD) at 730 nm.

For detection of osmolyte contents and mRNA levels, *Syn6803* cells were grown in column photo bioreactors (Tan et al., 2015) at 30°C with 5% CO_2_-air (v/v) aeration, and constant light illumination (100 μmol photons m^-2^ s^-1^) to the late exponential phase. Then, an aliquot BG11 supplemented with NaCl (5 M) was added to reach a final NaCl concentration of 684 mM (4%).

### RNA Isolation, Reverse Transcription PCR and Quantitative Real-Time PCR Analysis

Cells from 4 mL cultures with an OD_730_ of ∼5 were collected. Total RNA was isolated from cells using MiniBEST Universal RNA Extraction Kit (TaKaRa, Japan). The absence of genomic DNA from RNA preparations was verified by PCR using primer set rnpb-1 and rnpb-2 (Supplementary Table S4). Reverse transcription was done using PrimeScript^TM^ RT reagent Kit with gDNA Eraser (TaKaRa, Japan). Aliquots of the generated cDNA were used for the reverse transcription PCR (RT-PCR) assays. Quantitative Real-Time PCR was performed using SYBR Premix Ex Taq^TM^ II (TaKaRa) in a Light Cycler 480 (Roche). The primers shown in Supplementary Table S4 were used for real-time PCR.

### Determination of Intracellular Osmolytes in *Synechocystis* Cells

Cells from 2 ml aliquots of cultures were harvested by centrifugation at 12,000 × *g* for 5 min. The pretreatment method was designed according to our previous report (Du et al., 2013). Briefly, cells were suspended in 1 mL of 80% ethanol (v/v) and then incubated at 65°C for 4 h. After centrifugation at 12,000 ×*g* for 5 min, the supernatants were transferred into a clean tube and then dried under a stream of N_2_ at 55°C. The dry residues were dissolved in ultrapure water, subjected to membrane filtration (0.22 μm), and analyzed by ion chromatography ICS-5000 (Dionex, United States). The ion chromatography system was equipped with an electrochemical cell (Dionex, United States) and a Carb-Pacs^TM^ MA1 analytical column (4 mm × 250 mm). The column was equilibrated with 800 mM NaOH at a flow rate of 0.4 mL min^-1^.

### Enzymatic Activity Assay for Invertase

Sucrose degradation activity was determined according to the previously reported method (Vargas et al., 2003) with modifications. Reaction mixtures containing 100 mM potassium phosphate buffer (pH 7.0), 2 g L^-1^ sucrose and an appropriate volume of enzyme in a final volume of 50 μL were prepared. Mixtures were incubated at 30°C for 1.5 h. The amount of glucose was measured using ion chromatography. Proteins were quantified according to Bradford (Bradford, 1976).

## Results

### Reconstruction and Complementation of the *slr1588*-Null Mutant

**FIGURE 1:**
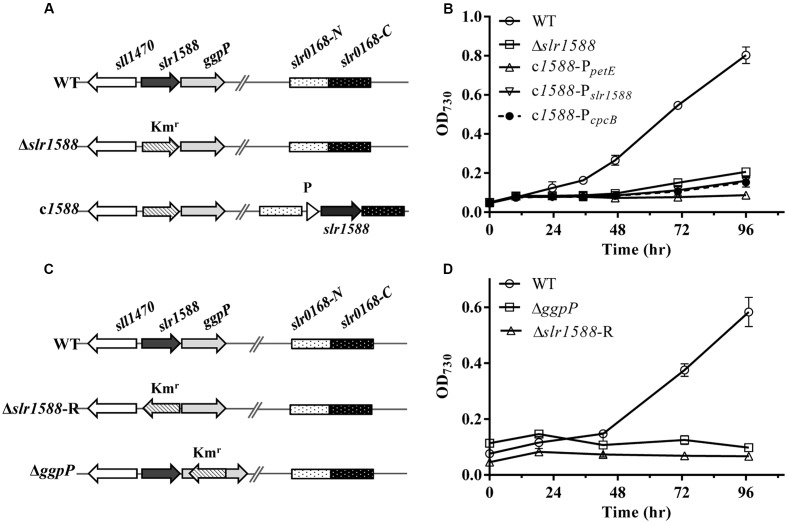
The genomic structures and growth of different strains of *Syn6803* under salt-stress condition. **(A)** Schematic representation of the *slr1588* and the *slr0168* loci in different strains. In the Δ*slr1588* mutant, the complete coding region of *slr1588* was replaced by a kanamycin resistance cassette (Km^r^). In the complementation strains, DNA fragments expressing the *slr1588* gene under control of three different promoters (P: P*_petE_*, P*_cpcB_* or P_*slr1588*_) were inserted into the neutral *slr0168* locus of the Δ*slr1588* mutant. **(B)** All five strains were grown in BG11 media containing 4% NaCl. Growth was monitored by measuring OD_730_. Error bars indicate standard deviation (*n* = 3). **(C)** In comparison to the initial Δ*slr1588* mutant, the Km^r^ fragment was inserted with reverse orientation in mutant Δ*slr1588*-R. The Km^r^ fragment replaced parts of the coding sequence in the Δ*ggpP* mutant. **(D)** All strains were grown in BG11 media containing 4% NaCl, and monitored by measuring OD_730_. Error bars indicate standard deviation (*n* = 3).

Mutants of *slr1588* showed different salt tolerance levels in previous reports (Hagemann et al., 1997b; Chen et al., 2014). To clarify whether or not the inactivation of Slr1588 was directly responsible for the salt-sensitive phenotype of the corresponding mutant of *Syn6803*, we initially constructed *slr1588* complementation strains in the *slr1588*-null mutant background. For this purpose, the *slr1588*-null mutant (Δ*slr1588*) was newly generated by replacing the complete coding sequence of *slr1588* with a kanamycin resistance cassette (**Figure 1A**), exactly as has been done in the previous study (Chen et al., 2014). Then, *slr1588* was ectopically expressed under control of three different promoters namely P*_petE_* (Tan et al., 2011), P*_cpcB_* (Zhou et al., 2014), or P*_*slr1588*_*, at a neutral site in the genome of the Δ*slr1588* mutant (**Figure 1A**).

The salt tolerance of the resulting strains was compared with the WT of *Syn6803*. The Δ*slr1588* mutant as well as all three complemented strains showed salt-sensitive phenotypes, i.e., they could not grow in the presence of 4% NaCl. These results indicate that the salt-sensitive phenotype of the Δ*slr1588* mutant is not directly related to the Slr1588 inactivation. Probably, there exists an additional mutation in Δ*slr1588* leading to its salt sensitivity as reported by Chen et al. (2014).

### Deletion of the Complete Coding Sequence of *slr1588* Disturbs the *ggpP* Expression

The disruption of the downstream situated gene *ggpP* resulted in salt sensitivity of *Syn6803* (Hagemann et al., 1997b), suggesting that polar effects of complete *slr1588* deletion on the expression of *ggpP* could be responsible for the salt sensitivity of the mutant Δ*slr1588*.

To prove this assumption, we first generated new mutants of *Syn6803*. Consistent with the previously reported *ggpP* mutant (Hagemann et al., 1997b), our newly generated Δ*ggpP* mutant, in which the kanamycin resistance cassette was inserted in reverse orientation into the *ggpP* gene (**Figure 1C**), showed the expected salt-sensitive phenotype (**Figure 1D**). Similarly, the newly generated Δ*slr1588*-R mutant, in which the kanamycin resistance gene was also inserted in opposite transcriptional orientation relative to *slr1588*, showed again the salt-sensitive phenotype (**Figures 1C,D**). Thus, the *ggpP* deletion resulted in the same salt-sensitive phenotype as the complete *slr1588* deletion.

**FIGURE 2:**
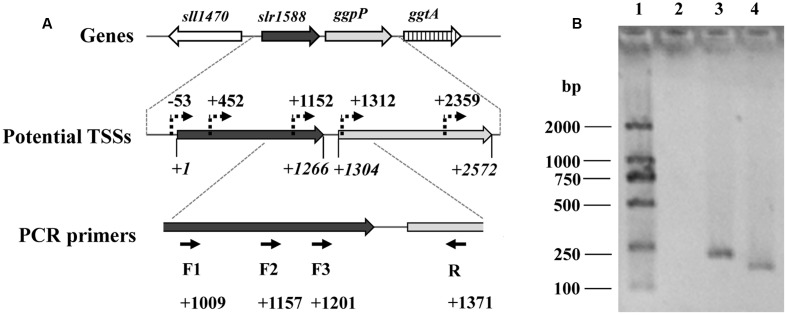
Analysis of the *ggpP* transcription by RT-PCR. **(A)**. The potential TSSs of *ggpP* inside *slr1588* predicted by dRNA-seq (Kopf et al., 2014) were labeled as dotted arrows. The translation start site of *slr1588* was set as +1, and the relative locations of these TSSs inside the ORF of *slr1588* or *ggpP* were calculated. For RT-PCR, three forward primers (F1, 2 and 3, binding sites inside slr1588 are indicated) specific to *slr1588* and a reverse primer (R) specific to *ggpP* were designed. **(B)**. Separation of DNA-fragments obtained by RT-PCR using cDNA of salt-grown cells of the wild type (WT) strain as template. Lane 1: a DNA ladder; Lane 2, 3 and 4: three primer pairs (F1/R, F2/R, and F3/R) were used for amplification, respectively.

We assumed that the *slr1588* mutation might somehow affect the *ggpP* expression. These two genes are only separated by 39 bp (Kaneko et al., 1996), but separately expressed. Recent dRNA-seq analyses (Kopf et al., 2014) mapped all transcriptional start sites (TSSs) in *Syn6803*. This study showed that three potential TSSs exist for *ggpP* transcription, two of them are located inside the *slr1588* coding sequence, namely at positions 452 and 1152, respectively (**Figure 2A**). To verify these TSSs of *ggpP*, we aimed to amplify DNA fragments overlapping *slr1588* and *ggpP* from cDNA of the WT strain grown under salt-stress condition. As shown in **Figure 2B**, these experiments verified the *ggpP* TSS at position 1152 inside *slr1588*, since PCR bands of the expected sizes were amplified using the primer pairs F2/R and F3/R but not with the primer pair F1/R. As positive controls, the expected bands could be obtained from genomic DNA of *Syn6803* using these primer pairs (Supplementary Figure S1). These results suggest that the transcripts of *ggpP* indeed overlapped with *slr1588*. However, the second intragenic TSS for *ggpP* at position 452 inside of *slr1588* could not be found here, possibly, it is not active under salt-stress conditions.

### Generation of *slr1588* Mutants Not Affecting *ggpP* Expression

**FIGURE 3:**
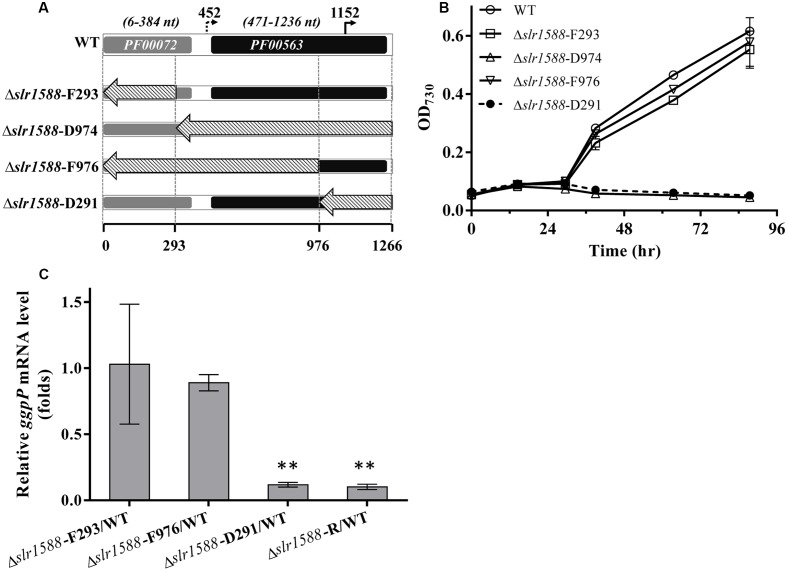
Salt tolerance and transcript levels of the *ggpP* gene in cells of mutants with partly deleted *slr1588*. **(A)** DNA fragments from positions 1 to 293, 294 to 1266, 1 to 976, and 977 to 1266 of *slr1588* coding sequence were replaced by the Km^r^ fragment resulting in the Δ*slr1588*-F293, D974, F976, and D291 mutants, respectively. **(B)** Growth curves of the four partly deleted *slr1588* mutants and the WT strain in BG11 supplemented with 4% NaCl. **(C)** *ggpP* transcript levels relative to WT cells in three *slr1588* mutants grown in BG11 supplemented with 4% NaCl for 24 h. The stars (^∗∗^) indicate significant differences (*p* < 0.05).

The above-described results make it likely that the deletion of the entire coding region of *slr1588* in the mutants Δ*slr1588* and Δ*slr1588*-R abolished *ggpP* expression leading to the salt-sensitive phenotype. Therefore, we aimed to obtain *slr1588* inactivation not interfering with *ggpP* transcription. To this end, we constructed four new *slr1588* mutants taking into consideration the domain structure of Slr1588 and the locations of the intragenic TSS of *ggpP* (**Figure 3**). According to the InterPro database, the Slr1588 protein consists of two domains, a receiving (pfam: 00072) and an EAL (pfam: 00563) domain. As shown in **Figure 3A**, these two domains are encoded in the sequence of *slr1588* from positions 6 to 384 and from positions 471 to 1236, respectively. These parts of the gene were separately deleted in the mutants Δ*slr1588*-F293 and Δ*slr1588*-D974, respectively. The two potential intragenic TSSs are located at positions 452 and 1152 of *slr1588*. Although we showed that the former TSS was inactive under salt-stress conditions, it might be important under other conditions. Based on these considerations, two additional mutants, Δ*slr1588*-F976 and Δ*slr1588*-D291 were generated, in which specific parts of the *slr1588* coding region were deleted but leaving the first or the second potential TSS of *ggpP* intact (**Figure 3A**).

The salt tolerance of these four *slr1588* mutants was compared to WT *Syn6803* grown in BG11 medium supplemented with 4% NaCl. Similar to the complete deletion mutant Δ*slr1588*-R, the Δ*slr1588*-D974 and Δ*slr1588*-D291 mutants showed no growth at high salt concentrations (**Figure 3B**). In all these three salt-sensitive mutants, the last 291 nts of the *slr1588* ORF including the second intragenic TSS of *ggpP* were deleted. In contrast, the Δ*slr1588*-F293 and the Δ*slr1588*-F976 mutants, where parts of coding sequence of *slr1588* and the first intragenic TSS of *ggpP* were deleted but the second TSS remained intact, showed the same salt tolerance as WT cells (**Figure 3B**).

The above-described results clearly indicate that not the absence of Slr1588 but the interference with *ggpP* expression is responsible for the salt sensitivity among these mutants. This conclusion was supported by qPCR analyses of *ggpP* in selected mutant strains under salt-stress conditions. As expected from the defined deletions, the *ggpP* transcription was significantly decreased in the Δ*slr1588*-D291 mutant, where the 3′ part of *slr1588* including the *ggpP* TSS at position 1152 was deleted. The *ggpP* mRNA content was ∼90% lower than in WT cells 24 h after salt shock (**Figure 3C**), similar to the level found in the Δ*slr1588*-R mutant (**Figure 3C** and **Table 1**). However, the *ggpP* transcript levels remained unchanged compared to WT in the two other mutants Δ*slr1588*-F293 and Δ*slr1588*-F976, where the TSS for *ggpP* at 3′ end of *slr1588* remained intact but 5′ parts of *slr1588* were deleted (**Figure 3C**). These results clearly support the notion that not the mutation of *slr1588* but the abolished *ggpP* transcription was responsible for salt-sensitive phenotype.

**Table 1:** Effects of gene deletion on transcript levels of genes encoding proteins involved in sucrose or glucosylglycerol (GG) metabolism.

Genes	Annotation	Δ*slr1588*-R/WT (folds)^a,b^	Δ*ggpP*/WT (folds)^a,b^	Δ*slr1588*-F976/WT (folds)^a,b^
*spsA*	Sucrose-phosphate synthase	1.863 ± 0.138^∗^	0.590 ± 0.031^∗^	1.747 ± 0.269^∗^
*spp*	Sucrose-phosphate phosphatase	5.965 ± 0.574^∗^	1.100 ± 0.042	1.298 ± 0.181
*ggpS*	Glucosylglycerol-phosphate synthase	2.037 ± 0.352	1.871 ± 0.043^∗^	0.814 ± 0.098
*ggpP*	Glucosylglycerol-phosphate phosphatase	0.102 ± 0.016^∗^	ND	0.890 ± 0.050
*invA*	Putative neutral invertase	0.388 ± 0.019^∗^	0.137 ± 0.021^∗^	1.106 ± 0.226
*slr1588*	Response regulator	ND	1.056 ± 0.257	ND

^a^*Data are shown as means ± SD (*n* = 3)*.

^b^*ND, Not determined*.

^∗^*Stars represent significant differences (*p* < 0.05) of gene transcription levels between the wild type (WT) and the mutant strains*.

### Effects of Specific Deletions of *slr1588* and *ggpP* on Gene Expression

In addition to salt tolerance, the Slr1588 protein was shown to be involved in the expression regulation of several genes encoding proteins involved of salt tolerance in *Syn6803* (Chen et al., 2014). Using defined mutants with defects in either *slr1588* or *ggpP*, we aimed to investigate the role of these proteins in gene expression regulation under salt-stress conditions. Thus, the single gene mutants Δ*slr1588*-F976 and Δ*ggpP* were compared with the Δ*slr1588-R* mutant (complete *slr1588* deletion and abolished *ggpP* expression) as well as WT of *Syn6803* regarding the transcript levels of *spsA*, *spp*, and *ggpS* in 24 h after salt shock (4% NaCl). As shown in the previous study (Chen et al., 2014), the mRNA levels of *spsA* and *ggpS* were significantly elevated in the Δ*slr1588-R* mutant (**Table 1**). However, the transcription levels of both, *ggpS* and *spp*, remained at WT levels in the Δ*slr1588*-F976 mutant (**Table 1**). Moreover, the expression level of *ggpS* was also similar in the mutants Δ*slr1588-R* and Δ*ggpP.* Only the gene *spsA* was significantly up-regulated in the Δ*slr1588*-F976 mutant (**Table 1**). These findings verify a specific role of Slr1588 as a negative regulator of *spsA* transcription under salt-stress condition in *Syn6803* as proposed by Chen et al. (2014), whereas the other transcriptional changes seem to be rather the result of the salt-sensitivity due to abolished *ggpP* expression in the Δ*slr1588*-R mutant.

### Specific Role of *slr1588* on Osmolyte Accumulation in *Syn6803*

**FIGURE 4:**
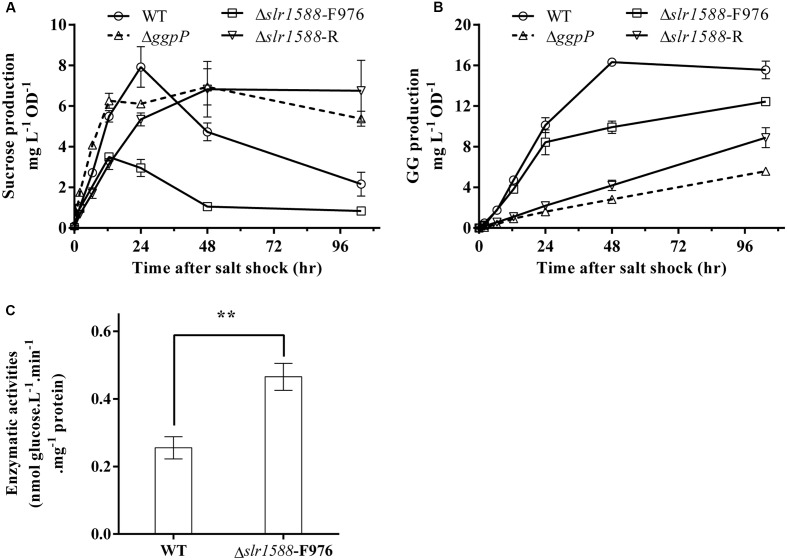
Effects of the *slr1588* inactivation on osmolyte contents and invertase. Cultures at late exponential phase of different strains were shifted to 4% of NaCl. Sucrose **(A)** and glucosylglycerol (GG) **(B)** contents were measured. **(C)** The invertase activity in the Δ*slr1588*-F976 was compared with that of WT in cells grown at 4% NaCl for 24 h. ^∗∗^Stars represent significant differences (*p* < 0.05) of enzymatic activities between the wild type (WT) and the Δ*slr1588*-F976 mutant strain.

Finally, we aimed to analyze whether or not the mutation of the Rre Slr1588 had specific effects on the amounts of sucrose and GG serving as main osmolytes in salt-stressed cells of *Syn6803* (Reed and Stewart, 1985). To this end, the mutants Δ*slr1588*-F976, Δ*slr1588*-R, Δ*ggpP*, as well as the WT were exposed to 4% NaCl up to 96 h. Sucrose contents increased to the peak level in first 24 h after salt shock. Subsequently, it decreased to only slightly elevated levels during the following 36 h. During this period, sucrose was replaced by GG that reached its maximum amount at about 48 h (**Figures 4A,B**). In the mutants Δ*slr1588*-R and Δ*ggpP* with impaired *ggpP* transcription, GG accumulation was strongly inhibited, whereas sucrose contents were kept unchanged after reaching their peak level (**Figures 4A,B**). These findings suggest that cells try to compensate for the decreased GG accumulation by enhanced sucrose accumulation as shown before for several mutants affected in GG synthesis (Marin et al., 1998). Surprisingly, we found only 44% of the sucrose content of WT cells in the Δ*slr1588*-F976 mutant despite the significantly enhanced *spsA* expression. GG accumulation rate was also slightly lower in this mutant compared to WT (**Figures 4A,B**). These findings indicate that the specific inactivation of Slr1588 without disrupting *ggpP* transcription leads to decreased osmolyte accumulation.

Post-transcriptional regulation of SpsA or stimulated sucrose breakdown by invertase are possible explanations for decreased sucrose amounts in mutant Δ*slr1588*-F976 despite stimulated *spsA* transcript amounts. To this end, we analyzed expression and activity of invertase, which is responsible for sucrose breakdown (Vargas et al., 2003). We did not find significant difference in the *invA* transcription between the WT and the Δ*slr1588*-F976 mutant under salt-stress conditions (**Table 1**), whereas *invA* expression was significantly reduced in the mutants Δ*ggpP* and Δ*slr1588*-R. However, the invertase activity in crude extracts of the mutant Δ*slr1588*-F976 was significantly higher than that in the WT (**Figure 4C**). This result partially explains why sucrose levels decreased after inactivation of Slr1588 in the presence of elevated *spsA* transcription.

## Discussion

Cyanobacterial acclimation to varying salt concentrations is well understood on the physiological and molecular level (Hagemann, 2011), but its sensing and signal transduction is only fragmentary known. Two-component systems are often involved in the response of bacteria to environmental factors. However, the systematic screening of Hik and Rre of *Syn6803* did not identify a system regulating osmolyte accumulation or overall salt tolerance (Marin et al., 2004; Shoumskaya et al., 2005). Similarly, the mutation of the Rre *orrA* abolished the salt-regulation of target genes without lowering the general salt or osmotic stress tolerance of *Anabaena* sp. PCC 7120 (Schwartz et al., 1998). Interestingly, Chen et al. (2014) reported that mutation of the Rre gene *slr1588* resulted in increased abundances of many salt-induced genes/proteins and was accompanied by a clear salt-sensitive phenotype. The latter finding is contrasting earlier findings, which reported unchanged salt tolerance of this mutant (Hagemann et al., 1997b). It is possible that differences in phenotypes could be owing to genome differences in laboratory strains of *Syn6803* (Ikeuchi and Tabata, 2001; Trautmann et al., 2012). However, observed sequence differences are rather low. And despite glucose utilization or changed motility, no clear phenotypic differences have been assigned to the genome sequence differences in these laboratory strain derivatives (Tajima et al., 2011). Thus, we assumed that possible polar effects of the different mutations could be responsible for these phenotypic differences, since the previous studies (Hagemann et al., 1997b; Chen et al., 2014) used different strategies for the *slr1588* knockout. Furthermore, these studies did not provide final proof of the linkage of mutation and phenotype by complementation approaches.

### The Salt-Sensitive Phenotype Is Linked to Abolished Expression of *ggpP* But Not of *slr1588* Mutation

To rule out strain differences, a new set of mutants were generated in the same WT background, i.e., the glucose-tolerant version of *Syn6803*. Initial complementation of the salt-sensitive phenotype by ectopic expression of *slr1588* in the newly generated deletion mutants Δ*slr1588* and Δ*slr1588*-R failed, i.e., these strain were still unable to grow at 4% NaCl (**Figure 1B**). Hence, polar effects of this mutation on other genes became likely, particularly on the downstream situated *ggpP* gene, whose mutation was linked with salt sensitivity (Hagemann et al., 1997b). Careful inspection of TSSs of *ggpP* provided by Kopf et al. (2014) and its experimental verification (**Figure 2**) indicated that the deletion of the complete *slr1588* also affected *ggpP* expression. This hypothesis was verified using mutants with only partial deletions of *slr1588*. For example, the deletion of the last 291 bp of *slr1588* resulted in a ∼90% decrease in *ggpP* transcription (**Table 1** and **Figure 3C**) linked with the expected salt sensitivity of these strains (**Figures 1B,D**, **3B**). These findings revealed that deletions of the 3′ part of *slr1588* also removed the promoter and the 5′-untranslated region (5′-UTR) of the downstream gene *ggpP*, thereby decreasing its expression as well as the salt tolerance of *Syn6803*.

### Role of Slr1588 on Transcription of *spsA*, *ggpS*, and *ggpP*

Chen et al. (2014) provided evidence that the changed expression of at least some salt-regulated genes resulted from direct binding of Slr1588 to the corresponding promoter regions. They showed a *spsA* upregulation, which was verified here using the newly generated Δ*slr1588*-F976 mutant, which is characterized by a partial deletion of *slr1588* leaving *ggpP* expression intact (**Figure 3C**). These findings support the notion of the previous report (Chen et al., 2014) that Slr1588 serves as a negative regulator for the *spsA* transcription.

However, the reported role of Slr1588 as a repressor of *ggpS* (Chen et al., 2014) could not be verified here. Consistent with the previous work we showed that the *ggpS* mRNA levels significantly increased in the complete *slr1588*-deletion mutant (**Table 1**). However, this alteration is clearly caused by the impaired *ggpP* transcription in this mutant, because *ggpS* transcription was increased by similar ratios in both, the Δ*slr1588*-R and Δ*ggpP* mutants (**Table 1**). In contrast, *ggpS* transcription was not affected in the Δ*slr1588*-F976 mutant, where *ggpP* transcription remained comparable to that of the WT strain (**Figure 3C**). Therefore, the single mutation of Slr1588 had no regulatory effect on the *ggpS* transcription of *Syn6803 in vivo* despite its binding to the *ggpS* promoter region *in vitro* as shown by Chen et al. (2014). Different potential regulators for *ggpS* have been identified in the last years. First, the small upstream-encoded protein GgpR was found to act as a repressor for *ggpS*, since its mutation resulted in enhanced *ggpS* expression (Klähn et al., 2010). Recently, the transcriptional factor LexA was identified to regulate many genes in *Syn6803*, among them it is also negatively affecting *ggpS* expression under low salt conditions (Klähn et al., 2010; Kizawa et al., 2016). Obviously, the *ggpS* gene is subject to a complex regulation involving different regulatory proteins of unknown hierarchical order. At least *in vivo*, the absence of Slr1588 was compensated by the other regulators leading to *ggpS* expression similar to WT.

### Role of Slr1588 on Accumulation of Sucrose and GG

Finally, we were interested to know whether expression changes of osmolyte genes correlate with product levels in the different mutant strains. Despite the clear increase of *spsA* transcript level, rather decreased sucrose amounts were found, when only Slr1588 was inactivated in the Δ*slr1588*-F976 mutant. Our previous report showed that the overexpression of *spsA* increased sucrose content under salt-stress condition (Du et al., 2013). However, in the Δ*slr1588*-F976 mutant, the inactivation of Slr1588 increased not only the *spsA* transcription (**Table 1**) but also the sucrose degradation activities (**Figure 4C**). The higher invertase activity provided a possible explanation for the decreased sucrose production in the Δ*slr1588*-F976 mutant. In addition, GG accumulation was also lower in cells of this mutant than in WT despite the similar mRNA levels of *ggpS* in these two strains (**Table 1**). GG accumulation is primarily controlled by the activity level of GgpS, which depends on contents of inorganic ions inside the cell (Novak et al., 2011). This biochemical regulation also explained that the GG contents remained almost unchanged in the *ggpR* mutant despite the increased abundance of GgpS (Klähn et al., 2010).

However, whether or not the Slr1588 plays a direct role for the regulation of the invertase activity or in the interplay of sucrose and GG biosynthesis with inorganic ion contents in *Syn6803* is not yet known. It is also possible that the primary osmolyte in *Syn6803*, GG, plays directly an important role in the regulation of salt tolerance processes. When GG biosynthesis was impaired by inhibiting the *ggpP* transcription, both the Δ*slr1588*-R and the Δ*ggpP* mutants showed increased sucrose levels to compensate for the absence of GG (**Figures 4A,B**) as has been reported before for the *ggpS* mutant (Marin et al., 1998). Considering the increased transcript levels of *ggpS*, *spsA*, and *spp* and the decreased *invA* levels in the Δ*ggpP* mutant (**Table 1**), it can be assumed that unbalanced GG and sucrose levels could lead to insufficient turgor pressure control, which may act as signals for the transcriptional as well as activity regulation of the involved genes/proteins. Future work is necessary to unravel the salt-sensing network and the specific function of the assigned transcriptional regulators such as GgpR, Slr1588, and LexA.

## Author Contributions

KS performed experiments, analyzed data and drafted the manuscript. XT designed the researches, analyzed data and drafted the manuscript. XL and MH analyzed data and drafted the manuscript. All authors read and approved the final manuscript.

## Conflict of Interest Statement

The authors declare that the research was conducted in the absence of any commercial or financial relationships that could be construed as a potential conflict of interest.
