# Supplementary material for: The Response Regulator Slr1588 Regulates spsA But Is Not Crucial for Salt Acclimation of Synechocystis sp. PCC 6803
Source: Front Microbiol. 2017 Jun 26;8:1176. doi: 10.3389/fmicb.2017.01176 (PMC5483463; doi:10.3389/fmicb.2017.01176)
Supplement: Supplementary file 1 [file Data_Sheet_1.docx]

**Supplementary Information for**

**The response regulator Slr1588 regulates *spsA* but is not crucial for salt acclimation of *Synechocystis* sp. PCC 6803**

Kuo Song^1^, Martin Hagemann^2^, Xiaoming Tan^1,*^, Xuefeng Lu^1,3,*^

^1^Key Laboratory of Biofuels, Shandong Provincial Key Laboratory of Synthetic Biology, Qingdao Institute of Bioenergy and Bioprocess Technology, Chinese Academy of Sciences, Qingdao, China

^2^Department of Plant Physiology, Institute of Biological Sciences, University of Rostock, Rostock, Germany

^3^Laboratory for Marine Biology and Biotechnology, Qingdao National Laboratory for Marine Science and Technology

^*^ **Correspondence:**

Xiaoming Tan: tanxm@qibebt.ac.cn;

Xuefeng Lu: lvxf@qibebt.ac.cn

**Table S1. Bacterial strains used in this study**

| **Strain name** | **Genotype description** *^a^* | **References** |
| --- | --- | --- |
| *Syn6803* | *Synechocystis* sp. PCC6803 wild type, glucose-tolerant | Prof. Xudong Xu |
| Δ*slr1588* | Δ*slr1588*::Km^r^ | This study |
| Δ*slr1588*-P*_petE_* | Δ*slr1588*::Km^r^ Δ*slr0168*::Omega P*_petE_-slr1588* | This study |
| Δ*slr1588*-P*_cpc_* | Δ*slr1588*::Km^r^ Δ*slr0168*::Omega P*_cpc_-slr1588* | This study |
| Δ*slr1588*-P*_slr1588_* | Δ*slr1588*::Km^r^ Δ*slr0168*::Omega P*_slr1588_-slr1588* | This study |
| Δ*slr1588*-R | Δ*slr1588*::R-Km^r^ | This study |
| Δ*slr0746* | Δ*slr0746*::R-Km^r^ (278^th^ to 523^rd^) | This study |
| Δ*slr1588*-F293 | Δ*slr1588*::R-Km^r^ (1^st^ to 293^rd^) | This study |
| Δ*slr1588*-D974 | Δ*slr1588*::R-Km^r^ (294^th^ to 1266^th^) | This study |
| Δ*slr1588*-F976 | Δ*slr1588*::R-Km^r^ (1^st^ to 976^th^) | This study |
| Δ*slr1588*-D291 | Δ*slr1588*::R-Km^r^ (977^th^ to 1266^th^) | This study |

*^a^* P_petE_, the promoter of *petE* gene. P_cpc_, the promoter of *cpc* operon. P_slr1588_, 0.2 Kb DNA fragment upstream of *slr1588* gene. Km, a kanamycin resistant cassette. Omega, a spectinomycin resistant cassette. R-Km^r^, a kanamycin resistant cassette was inserted into the *slr1588* ORF with a reverse orientation. Numbers in the brackets indicate this part of the *slr1588* ORF is replaced by a Km^r^ cassette.

**Table S2. Plasmids used and constructed in this study**

| Plasmid name | Functions | References |
| --- | --- | --- |
| pSK005 | For expression *slr1588* with the P*_petE_* promoter in the *slr0168* locus | This study |
| pSK007 | For deletion of the complete ORF of *slr1588* | This study |
| pSK008 | For expression *slr1588* with the P*_cpc_* promoter in the *slr0168* locus | This study |
| pSK013 | For expression *slr1588* with the P*_slr1588_* promoter in the *slr0168* locus | This study |
| pSK023 | For replacing the complete ORF of *slr1588* with a reverse Km^r^ fragment | This study |
| pSK058 | For replacing the 1^st^ to 293^rd^ nts of the *slr1588* ORF with a reverse Km^r^ fragment | This study |
| pSK059 | For replacing the 294^th^ to 1266^th^ nts of the *slr1588* ORF with a reverse Km^r^ fragment fragment of *slr1588* ORF | This study |
| pSK060 | For replacing the 1^st^ to 976^th^ nts of the *slr1588* ORF with a reverse Km^r^ fragment | This study |
| pSK061 | For replacing the 977^th^ to 1266^th^ nts of the *slr1588* ORF with a reverse Km^r^ fragment | This study |
| pSK073 | For replacing the 278^th^ to 523^rd^ nts of the *ggpP* ORF with a reverse Km^r^ fragment | This study |

**Table S3. Primers used for construction of plasmids**

| Primer names | Primer sequences (5’-3’) | Description |
| --- | --- | --- |
| pSK005-1 | CGCCATATGAGTACTATTTTAGTTGTCGAG | Ligation of *slr1588* into pXT37b, for construction of pSK005 |
| pSK005-2 | CCGCTCGAGTCAGCGCCGTAGGGGCAAAATAG | Ligation of *slr1588* into pXT37b, for construction of pSK005 |
| Km-1 | AGCGAGGTATGTAGGCGGTGCT | Amplification of Km^r^ fragment from pET28a for fusion Km^r^ with different homologous arms |
| Km-2 | GGAAATGTGCGCGGAACCCCT | Amplification of Km^r^ fragment from pET28a for fusion Km^r^ with different homologous arms |
| pSK007-1 | CCGGCTCACTGATGCCAATGCCTTGGC | Fusion *slr1588* arms with Km^r^, for construction of pSK007 |
| pSK007-2 | ACTCTGTAGCACCGCCTACATACCTCGCTAGGGATTAATCAAATCTTTG | Fusion *slr1588* arms with Km^r^, for construction of pSK007 |
| pSK007-3 | AAACAAATAGGGGTTCCGCGCACATTTCCCAGTTCAGTCAAGATGCGAA | Fusion *slr1588* arms with Km^r^, for construction of pSK007 |
| pSK007-4 | GTCCACATACACCACTAAGTTACCGTG | Fusion *slr1588* arms with Km^r^, for construction of pSK007 |
| pSK007-F | GCCACCATTGCACTGCCTTGT | Nest primer, for construction of pSK007 |
| pSK007-R | CGCTCCCGCCACGTCGAAT | Nest primer, for construction of pSK007 |
| pSK013-1 | GGTACCGGTGGAAGTCTAGACAGGGCGATCGCCGAG | Ligation of *slr1588* into pXT37b, for construction of pSK013 |
| pSK013-2 | CTCGAGGAACTGTCAGCGCCGTAGGGGCAAAATAGGAAC | Ligation of *slr1588* into pXT37b, for construction of pSK013 |
| pSK023-1 | Same as pSK007-1 | Fusion *slr1588* arms with Km^r^, for construction of pSK023 |
| pSK023-2 | TTCGCATCTTGACTGAACTAGTAGGGATTAATCAAATCTTTG | Fusion *slr1588* arms with Km^r^, for construction of pSK023 |
| pSK023-3 | CAAAGATTTGATTAATCCCTACTAGTTCAGTCAAGATGCGAA | Fusion *slr1588* arms with Km^r^, for construction of pSK023 |
| pSK023-4 | Same as pSK007-4 | Fusion *slr1588* arms with Km^r^, for construction of pSK023 |
| pSK023-F | Same as pSK007-F | Nest primer, for construction of pSK023 |
| pSK023-R | Same as pSK007-R | Nest primer, for construction of pSK02 |
| pSK058-1 | CATCTGGGTTGATATAGCCGCAGCGAGC | Fusion *slr1588* arms with Km^r^, for construction of pSK058 |
| pSK058-2 | AGGGGTTCCGCGCACATTTCCAGGGATTAATCAAATCTTTG | Fusion *slr1588* arms with Km^r^, for construction of pSK058 |
| pSK058-3 | AGCACCGCCTACATACCTCGCTCGGCGCGGATGATTACTTAAT | Fusion *slr1588* arms with Km^r^, for construction of pSK058 |
| pSK058-4 | GGTGAAATCTGCCACTGGCAGAGGACG | Fusion *slr1588* arms with Km^r^, for construction of pSK058 |
| pSK058-F | CCTCAATGGACATATTACAAACGGTCATG | Nest primer, for construction of pSK058 |
| pSK058-R | GATAGCCTTGTAACTCGTCACACCCCATG | Nest primer, for construction of pSK058 |
| pSK059-1 | Same as pSK58-1 | Fusion *slr1588* arms with Km^r^, for construction of pSK059 |
| pSK059-2 | AGGGGTTCCGCGCACATTTCCGAATTCATGCCCTTACGAAAATC | Fusion *slr1588* arms with Km^r^, for construction of pSK059 |
| pSK059-3 | AGCACCGCCTACATACCTCGCTCAGTTCAGTCAAGATGCGAAA | Fusion *slr1588* arms with Km^r^, for construction of pSK059 |
| pSK059-4 | Same as pSK58-4 | Fusion *slr1588* arms with Km^r^, for construction of pSK059 |
| pSK059-F | Same as pSK58-F | Nest primer, for construction of pSK059 |
| pSK059-R | CAATGATCAGCGGCATCAAGGCCGGATC | Nest primer, for construction of pSK059 |
| pSK060-1 | Same as pSK58-1 | Fusion *slr1588* arms with Km^r^, for construction of pSK060 |
| pSK060-2 | Same as pSK58-2 | Fusion *slr1588* arms with Km^r^, for construction of pSK060 |
| pSK060-3 | AGCACCGCCTACATACCTCGCTCTGCAGCAGTTCCCCTTTGATG | Fusion *slr1588* arms with Km^r^, for construction of pSK060 |
| pSK060-4 | Same as pSK59-4 | Fusion *slr1588* arms with Km^r^, for construction of pSK060 |
| pSK060-F | Same as pSK58-F | Nest primer, for construction of pSK060 |
| pSK060-R | Same as pSK59-R | Nest primer, for construction of pSK060 |
| pSK061-1 | GTCGAGGATGAAGCCATTATTCGGGAG | Fusion *slr1588* arms with Km^r^, for construction of pSK061 |
| pSK061-2 | AGGGGTTCCGCGCACATTTCCATAACTTAGGGAAGAGTAACCAG | Fusion *slr1588* arms with Km^r^, for construction of pSK061 |
| pSK061-3 | Same as pSK59-3 | Fusion *slr1588* arms with Km^r^, for construction of pSK061 |
| pSK061-4 | Same as pSK59-4 | Fusion *slr1588* arms with Km^r^, for construction of pSK061 |
| pSK061-F | GGCGAAACCCTGAGTCTGGAAAATTATAAG | Nest primer, for construction of pSK061 |
| pSK061-R | Same as pSK59-R | Nest primer, for construction of pSK061 |
| pSK073-1 | GGCTGTGAAGCCTTGATCCGTTGGCACCATCCC | Fusion *ggpP* arms with Km^r^, for construction of pSK073 |
| pSK073-2 | GGGGTTCCGCGCACATTTCCCCTGTTGCACAAAGGAAGCATC | Fusion *ggpP* arms with Km^r^, for construction of pSK073 |
| pSK073-3 | GCACCGCCTACATACCTCGCTCCTTGGCCAATCTGTTACAAGAC | Fusion *ggpP* arms with Km^r^, for construction of pSK073 |
| pSK073-4 | CCCGGTCTTTGGGAGATAGTTCATTCACCCGG | Fusion *ggpP* arms with Km^r^, for construction of pSK073 |
| pSK073-F | CTGGCTTTATCAGCGTGGTGGGG | Nest primer, for construction of pSK073 |
| pSK073-R | GCCAATGCAGATTTGTCCTTGAC | Nest primer, for construction of pSK073 |

**Table S4. Primers used for qRT-PCR.**

| Primer names | Primer sequences (5’-3’) | Description |
| --- | --- | --- |
| sll0045-F | GAGGATAATAGGCTTACGGG | Amplification of small fragment of *sll0045* |
| sll0045-R | CATCAGGAAATCGCAGAAC | Amplification of small fragment of *sll0045* |
| slr0953-F | AACGGAGATGTTGAAGGAA | Amplification of small fragment of *slr0953* |
| slr0953-R | ACACCAGGGTTTGAGACG | Amplification of small fragment of *slr0953* |
| sll1556-F | AGTGATCCAGGCAATGTCG | Amplification of small fragment of *sll1556* |
| sll1556-R | AGGGAATGCGTATTTATCG | Amplification of small fragment of *sll1556* |
| slr0746-F | AGGTAGGGATGAACGAGG | Amplification of small fragment of *slr0746* |
| slr0746-R | GCTTGGCGAGCACTAAA | Amplification of small fragment of *slr0746* |
| rnpb-F | TGAGGACAGTGCCACAGAA | Amplification of small fragment of *rnpb* |
| rnpb-R | AATTCCTCAAGCGGTTCCAC | Amplification of small fragment of *rnpb* |

Figure S1. Evaluation of primers used for RT-PCR. For positive controls, the genomic DNA of *Synechocystis* sp. PCC 6803 was used as a template. Lane 1: a DNA ladder; Lane 2, 3 and 4: three primer pairs (F1/R, F2/R, and F3/R) were used for PCR amplification respectively. The clear PCR bands show that primers are valid for detecting transcripts by RT-PCR.
